# Influences of reopening businesses and social venues: COVID-19 incidence rate in East Texas county

**DOI:** 10.1017/S0950268821000121

**Published:** 2021-01-18

**Authors:** Tuan D. Le, Michele Bosworth, Gerald Ledlow, Tony T. Le, Jeffrey Bell, Karan P. Singh

**Affiliations:** 1 Department of Epidemiology and Biostatistics, School of Community and Rural Health, The University of Texas Health Science Center at Tyler, Tyler, Texas, USA; 2 U.S. Army Institute of Surgical Research, JBSA-Fort Sam Houston, San Antonio, Texas, USA; 3 Center for Population Health, Analytics and Quality Advancement, School of Community and Rural Health, The University of Texas Health Science Center at Tyler, Tyler, Texas, USA; 4 Department of Healthcare Policy, Economics and Management, School of Community and Rural Health, The University of Texas Health Science Center at Tyler, Tyler, Texas, USA; 5 Department of Family Medicine, The University of Texas Health Science Center at Tyler, Tyler, Texas, USA

**Keywords:** COVID-19, economic reopening, rural health, SARS-CoV-2

## Abstract

As the on-going severe acute respiratory syndrome coronavirus 2 pandemic, we aimed to understand whether economic reopening (EROP) significantly influenced coronavirus disease 2019 (COVID-19) incidence. COVID-19 data from Texas Health and Human Services between March and August 2020 were analysed. COVID-19 incidence rate (cases per 100 000 population) was compared to statewide for selected urban and rural counties. We used joinpoint regression analysis to identify changes in trends of COVID-19 incidence and interrupted time-series analyses for potential impact of state EROP orders on COVID-19 incidence. We found that the incidence rate increased to 145.1% (95% CI 8.4–454.5%) through 4th April, decreased by 15.5% (95% CI −24.4 −5.9%) between 5th April and 30th May, increased by 93.1% (95% CI 60.9–131.8%) between 31st May and 11th July and decreased by 13.2% (95% CI −22.2 −3.2%) after 12 July 2020. The study demonstrates the EROP policies significantly impacted trends in COVID-19 incidence rates and accounted for increases of 129.9 and 164.6 cases per 100 000 populations for the 24- or 17-week model, respectively, along with other county and state reopening ordinances. The incidence rate decreased sharply after 12th July considering the emphasis on a facemask or covering requirement in business and social settings.

## Introduction

Coronavirus disease 2019 (COVID-19) caused by severe acute respiratory syndrome coronavirus 2 (SARS-CoV-2) is an on-going pandemic. The first COVID-19 case in the U.S. was detected on 20 January 2020 in Washington State and was traced to Wuhan, China where initial cases were reported [1, 2]. COVID-19 cases have increased exponentially with human-to-human transmission mode in close unprotected contacts [2, 3]. As of 29 August 2020 it has infected more than 25 million people and killed more than 843 thousand patients globally [4]. In the U.S., approximately 6 million confirmed cases and more than 182 thousand deaths have been reported since the first U.S. COVID-19 outbreak [4].

Non-pharmaceutical interventions such as early detection and quarantines, travel bans or movement management and restriction on public gatherings, have been reported as effective strategies to prevent or reduce COVID-19 transmission for ‘flatten the curve’ of the pandemic in the outbreak in China and other countries at risk globally [3, 5–7].

Social distancing has been implemented in most states since March 2020 and was found to decrease the spread of SARS-CoV-2 [8–11]. However, a two-phased reopening of economies was implemented in most states in mid-May 2020, leading to lifting of social distancing requirements that impacted COVID-19 incidence rates [12, 13]. Reopening businesses during the coronavirus (COVID-19) pandemic poses a challenge due to the need for widespread containment and resurgence mitigation strategies [12, 14]. However, influence of each phased ‘economic reopening’ on COVID-19 incidence rates along with influence of other events such as those that prompt large gatherings (‘Black Lives Matter’ protests and 4th July weekend celebrations) along with mask mandates have not been evaluated.

The changes of social, political, health and financial activities provide the context of impact on community concordance or attention to guidance intended to reduce the virus spread. It is impossible to remove the fabric of life from the pandemic; decisions are made by individuals, groups, communities and society as a whole in that pluralistic reality. The goal of this study was to analyse pandemic incidence within the construct of community concordance. A simple definition of the construct is

Community Concordance = environmental factors (includes policy, law, enforcement, social norms, social issues etc…) + discipline/fatigue + group adherence to non-pharmaceutical mitigation or interventions + individual adherence to non-pharmaceutical mitigation or intervention. Therefore, higher community concordance should reduce COVID-19 infection rates, whereas less concordance increases infection loads. According to well-published CDC guidance, non-pharmaceutical interventions include: (1) social distancing of six feet minimum; (2) social load (density) that negates larger group gatherings; (3) individual utilisation of face coverings and (4) individual responsibility for frequent hand hygiene according to CDC guidance.

Disproportionally, COVID-19 studies have been conducted in large populations such as multiple countries, country and urban populations [15], but not a rural or semi-rural region. Therefore, in this study we examined trends and influence of policies or non-pharmaceutical interventions on trends of COVID-19 incidence in a semi-rural county, Smith County, with comparisons to statewide trends of Texas and other selected urban and rural counties: Dallas, Tarrant, Denton, Travis, Harris, Bexar and Galveston. Smith County, a semi-rural county in Northeast Texas, reported its first COVID-19 case on 13 March 2020 [16].

## Methods

### Patient population

The study was a population-based county-level time-series analysis of all residents of Smith County, Texas between 15 March 2020 and 29 August 2020. Smith County is basically rural with a small city, Tyler, in the centre of the county; semi-rural would be a reasonable designation. Publicly available data on COVID-19 consisting of the total number of cases, recovered cases and dead cases for Smith County and other comparative counties were extracted from the websites of the Texas Health and Human Services (DSHS) COVID-19 Data [17] and standardised new confirmed COVID-19 cases to county population using the U.S. Census Bureau data for Smith County with an estimated population of 232 751 persons based on U.S. Census on 1 July 2019 [18].

This study was exempted from review by the institution review board of the University of Texas Health Science Center at Tyler, Texas, because the data for this analysis was obtained from a publicly available source.

### Study definitions

Economic reopening phase 1 on 1 May 2020 (EROP1; week 8) [19, 20] and phase 2 between 20 May 2020 and 24 May 2020 (EROP2; week 11) [21, 22] are used as independent variables to investigate whether these policies influenced trends in COVID-19 incidence during the COVID-19 pandemic period from initiation until 29 August 2020.

The primary measure of interest was the incidence rate of the COVID-19 defined as number of COVID-19 cases per 100 000 person-week between 15 March 2020 and 29 August 2020 (24 weeks) with numerators defined by the number of new COVID-19 cases in the respective calendar weeks and denominators by the total population at risk in the corresponding week. The incidence rate reflects all ages of the 2019 U.S. standard population.

### Statistical analysis

Data on COVID-19 were used to describe COVID-19 incidence and trends in a semi-rural county and compared to other rural and urban counties in Texas during the pandemic period. Descriptive analysis was performed using chi-square test for association of demographics and COVID-19 distributions. We graphed the average number of new COVID-19 cases daily and weekly; with an estimated incidence rate by gender, race and age groups in the Smith County population. We also performed visual comparison trajectories in incidence rates between Smith County, other counties and Texas as aggregate.

Temporal trends in COVID-19 incidence rate were evaluated using a joinpoint regression analysis (Joinpoint Trend Analysis Software version 4.8.0.1, 22 April 2020; Surveillance, Epidemiology and End Results Program, National Cancer Institute) [23]. Joinpoint regression was used to calculate the weekly percentage change (WPC) and fitted to estimate average weekly changes (AWPC) to identify joinpoints at which significant changes in trends in COVID-19 incidence occurred during the pandemic period studied. The logarithmic transformation of the COVID-19 incidence was applied and the heteroscedastic errors option was assumed as standard errors. We also estimated 95% confidence intervals (CIs) for each estimate of WPC and AWPC and investigated whether the WPC for each segment and AWPC for overall differed significantly from zero.

To assess the change in COVID-19 incidence, we conducted an interrupted time-series analysis (ITSA). It is useful and well-known as a simple quasi-experimental design used for evaluating the effect of a defined intervention on an outcome of interest when randomised control trial data are not available. ITSA was applied using the following segmented regression model for the outcome defined as trends in incidence of COVID-19 after each intervention [EROP 1 (estimated as week 8 of 3 May–9 May 2020) and EROP 2 (estimated week 12 as week of 31 May–6 June 2020)] and their synergistic effect. For effect of each intervention, the segmented regression analyses were as adapted [24, 25]. Influence of EROP 1 or EROP 2 on COVID-19 incidence is described as a change in the level and slope as follows: For single intervention, EROP 1, COVID-19 incidence rate is
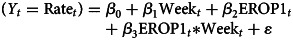
to evaluate influence of independent variable (*X_i_*) EROP1 on trend in the COVID-19 incidence after each phase implemented. The *Y_t_* is the outcome. The *β*_0_ represents an intercept or starting level where Time = 0, *β*_1_ is a change in COVID-19 incidence associated with an increase of each week in pre-intervention. The *β*_2_ is the level change after EROP1 implemented, and *β*_3_ indicates the slope change or the change in trend between the implementation of EROP1 and EROP2. The post-intervention trend after introducing of EROP 1 or absolute effect of a single intervention of the outcome is calculated as *β*_2_ + *β*_3_. *ε* is a random error.

For additional intervention of EROP 2, the above equation is modified to calculate effect of each single and synergistic effect of EROP 1 and EROP 2 accordingly as follows:

COVID-19 rate = *β*_0_ + *β*_1_Week_t_ + *β*_2_EROP1_t_ + *β*_3_EROP1_t_ *Week_1t_ + *β*_4_EROP2_t_ + *β*_5_EROP2_t_ *Week_2t_ + *ε*_t_.

The *β*_4_EROP2_t_ + *β*_5_EROP2_t_*Week_2t_ represent the additional intervention (EROP2). Thus, *β*_4_ represents change in level of COVID-19 rate immediately following the EROP 2, and *β*_5_ indicates the difference slopes of COVID-19 rate between the EROP1 and the EROP 2. *β*_1_ indicates the change in COVID-19 incidence rate per week in the pre-intervention segment; the difference trend between pre-EROP 1 and the EROP 1 trend (*β*_3_); the difference trend between the EROP 1 and EROP 2 (*β*_5_); the EROP 1 trend was calculated as (*β*_1_ _+_ *β*_3_); the EROP 2 trend was calculated as (*β*_1_ _+_ *β*_3_ _+_ *β*_5_); the difference between the EROP 2 and the pre-EROP 1 trend was calculated as (*β*_3 +_ *β*_5_).

To estimate the effect of the synergistic effect of the two interventions, we estimated absolute effect of these EROP(s) in two models: model 1 for the entire study period (15 March–29 August 2020) and model 2 for the study period up to week of 17 (15 March–11 July 2020) when the COVID-19 incidence reaches a peak at week 17 (4 July–11 July 2020). Cumby−Huizinga test was also used for autocorrelation and lag = 1 was defined. The series had a unit root defined as more than 1 trend in the series by using an augmented Dickey−Fuller test. Statistical significance was determined at the *P* < 0.05 level. Analyses were conducted in STATA version 16 (StataCorp LP, College Station, TX, USA).

## Results

### Patient population

Between 15 March 2020 and 29 August 2020, 3645 COVID-19 cases, including 3207 confirmed cases were reported, accounting for 1.6% of the Smith County population or cumulative incidence rate of 1566.1 (confirmed + probable) or 1377.9 (confirmed) cases per 100 000 residents (Table 1). A higher incident rate of COVID-19 cases was observed in females than males, 1446.4 vs. 1304.4 cases per 100 000 persons (*P* < 0.001). Highest incidence rate of COVID-19 was observed in Hispanic (2385.5 cases), followed by Black (1617.2 cases), then White (1000.3 cases) and Asian population (778.7 cases) per 100 000 person-week (*P* < 0.001; Table 1). Patient age ranged from 0 to 100 years with higher COVID-19 cases in the age group of 21–60 (*n* = 2005), accounting for 62.5% of total contracted SARS-CoV-2 cases in this county and incidence rate of 1731.8 cases per 100 000 person-week, followed by age group of 10–19 years, then age of ≥60 years and age of 0–9 (*P* < 0.001; Table 1).

**Table 1.** Overall demographics and COVID-19 patient characteristics

	Smith County	COVID-19 cases^a^	Incidence rate	*P*-value
No. (%)	232 751b	3645 (1.57)	1566.1	–
Confirmed	232 751	3207 (1.38)	1377.9	–
Probable	232 751	438 (0.19)	188.2	–
Recovered, no. (%)				
All	3645	2764	75.8	–
Confirmed	3207	2481	89.8	–
Probable	438	283	10.2	–
Deaths, no. (%)				
All	–	51	1.4	–
Confirmed	–	47	1.5	–
Probable	–	4	0.9	–
Among contracted SARS-CoV-2 (confirmed cases *N* = 3207)				
Gender, no. (%)				<0.0001
Male	112 316 (48.3)	1465 (40.2)	1304.4	
Female	120 435 (51.7)	1742 (47.8)	1446.4	
Race/Ethnicity, no. (%)^c^^,^^d^				<0.0001
White	137 556 (59.1)	1376 (42.9)	1000.3	
Black	41 430 (17.8)	670 (20.9)	1617.2	
Hispanic	46 783 (20.1)	1116 (34.8)	2385.5	
Asian	3724 (1.6)	29 (0.9)	778.7	
Other	3258 (1.4)	16 (0.5)	491.1	
Age, year, no. (%)				<0.0001
0–9	31 332 (13.5)	160 (5.0)	510.7	
10–19	32 314 (13.9)	404 (12.6)	1250.2	
20–59	115 777 (49.7)	2005 (62.5)	1731.8	
≥60	53 328 (22.9)	638 (19.9)	1196.4	

COVID-19, Coronavirus Disease 2019.

a Data was abstracted from Texas Demographics.

b COVID-19 data were abstracted from Texas Health and Human Service and Net Health Northeast Texas Public Health District.

c Estimated race/ethnicity numbers based on U.S. Census for Smith County of Texas from https://www.census.gov/quickfacts/fact/table/smithcountytexas; accessed on 1 October 2020.

d Estimated COVID-19 cases by race/ethnicity based on distribution of COVID-19 cases from patients with documented race/ethnicity abstracted from https://nethealthcovid19.org.

Approximately 76% of patients recovered from contracted COVID-19 (*n* = 2764) with an overall death rate of 1.4% (*n* = 51).

### Distribution of COVID-19 incidence and its weighted moving average trend

**Fig. 1.**
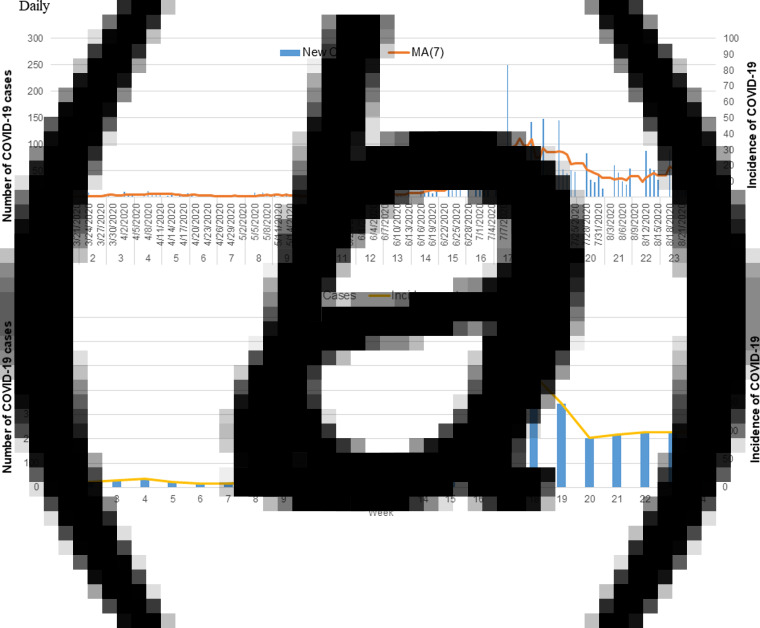
New COVID-19 cases and incidence rate per 100 000 population of the Smith County daily (panel A) and weekly (panel B). Left vertical axis represents number of COVID-19 cases. Right vertical axis represents incidence rate of COVID-19.

The trends of observed daily and weighted 7-day moving average of confirmed COVID-19 cases (*n* = 3207) during the study period are depicted in Figure 1a. Daily confirmed number of cases increased since 7 June, reached a peak of 250 cases on 8 July, then declined. The daily cumulative weighted moving average curve also showed that the COVID-19 did not increase until early June then peaked to 37.23 cases per 100 000 population on 11 July then declined. Similarly, weekly COVID-19 cases and its corresponding trend demonstrated that COVID-19 cases and its trend began to increase sharply in the week of 31 May–6 June 2020, reached a peak of 250 cases per 100 000 person-week in the week of 5 July–11 July 2020 and then declined (Fig. 1b).

### Joinpoint regression analysis for trend in the COVID-19 incidence rate

**Fig. 2.**
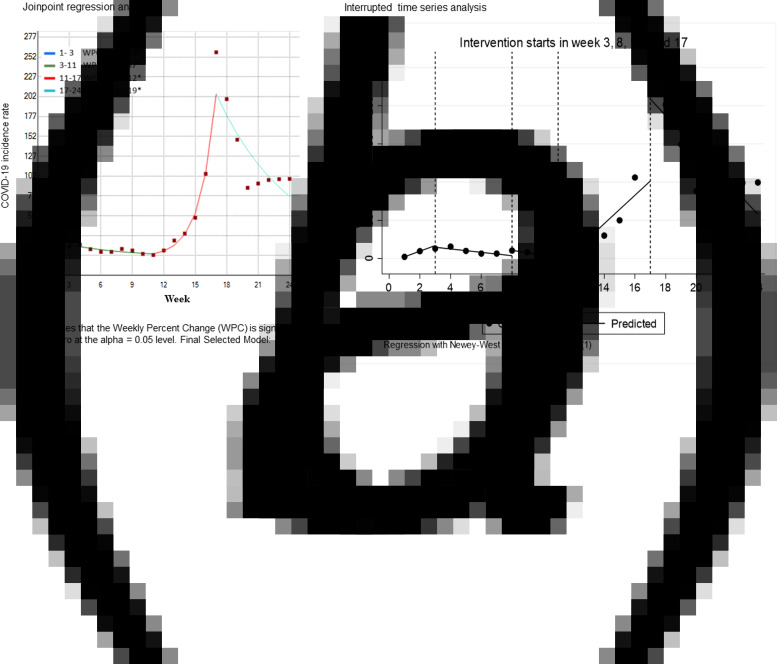
Incidence rate illustrated trends in changes of COVID-19 incidence and points of WPC were estimated. Joinpoint regression model revealed three joinpoints in weeks 3, 11 and 17 since 15 March 2020 at which trends in COVID-19 incidence significantly changed and defined pandemic study period was segmented into four periods: 15 March–4 April 2020 (weeks 1−3), 5 April–30 March 2020 (weeks 4−11), 31 May–11 July 2020 (weeks 12−17) and 12 July–29 August 2020 (weeks 18−24) with WPC (95% CI) from zero (panel A). Panel B depicted interrupted time series with COVID-19 incidence trends with level changes.

Overall, COVID-19 incidence trend significantly increased with an AWPC of 15.9% (95% CI 5.9–26.8%) (Table 2 and Fig. 2). The joinpoint regression model revealed three joinpoints in weeks 3, 11 and 17 since 15 March 2020 at which trends in COVID-19 incidence significantly changed (Fig. 2a); therefore, the defined pandemic study period was segmented into four periods: 15 March–4 April 2020 (weeks 1–3), 5 May–30 May 2020 (weeks 4–11), 31 May–11 July 2020 (weeks 12–17) and 12 July–29 August (weeks 18–24) with WPC (95% CI) from zero. The trend first increased from week 1 (2.1 cases per 100 000) to week 3 (12.9 cases per 100 000) with a WPC of +145.1% (95% CI 8.4–454.5%), followed by a slight decrease from week 4 (15.5 cases per 100 000) to week 11 (2.6 cases per 100 000) with a WPC of −15.7% (95% CI −24.4, −5.9%). Incidence increased again sharply to reach a peak of 257 cases per 100 000 population – in week 17 (5 July–11 July 2020) with a WPC of +93.1% (95% CI 60.9–131.8%) and then declined significantly to the end of study period in week 24 (98.4 cases per 100 000) with a WPC of −13.2% (95% CI −22.2, −3.2%) (Table 2).

**Table 2.** WPC in COVID-19 incidence according to joinpoint regression

Trend	Period (weeks)	Date	WPC (95% CI)**
1	1–3	3/15–4/4	145.1 (8.4454.5)*
2	3–11	4/5–5/30	−15.7 (−24.4, −5.9)*
3	11–17	5/31–7/11	93.1 (60.9–131.8)*
4	17–24	7/12–8/29	−13.2 (−22.2, −3.2)*
Overall (AWPC)	1–24	3/15–8/29	15.9 (5.9–26.8)*,**

CI, confidence interval; AWPC, average weekly percentage change.

*Indicates that the WPC is significantly different from zero at the two-sided *P* < 0.05 level.

**Indicates that the AWPC is significantly different from zero at the two-sided *P* < 0.05 level.

### Association of economic reopening of business and social venues implementation and changes in COVID-19 incidence

**Fig. 3.**
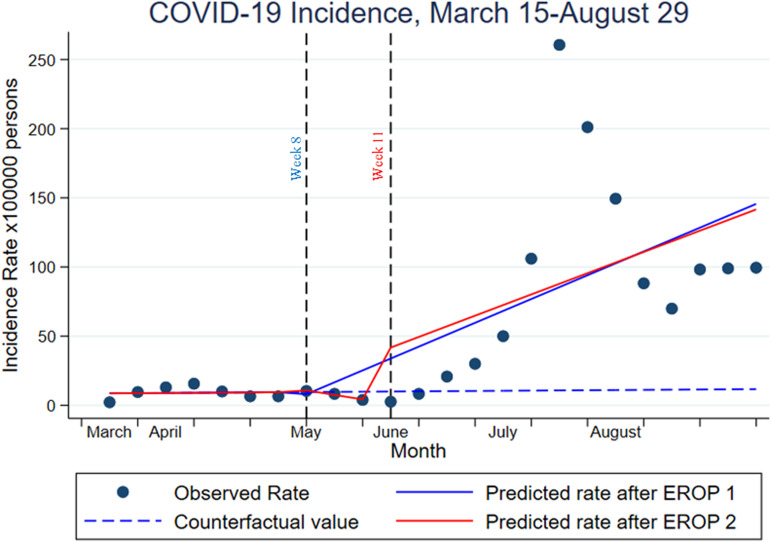
Impact of reopening business on COVID-19 depicted incidence rate of COVID-19 with observed rate (black dots), estimated counterfactual trend of COVID-19 if economic reopening business (EROP) did not happen (dash blue line), predicted trend after EROP 1 implemented (solid blue line) and predicted trend after EROP 2 implemented (solid red line). Vertical lines at weeks 8 and 11 where the EROP 1 and EROP 2 started.

There were significant differences in absolute COVID-19 cases and COVID-19 incidence rate between before and after EROP 1 and EROP 2 phases with means of 20.9 vs.176.9 *vs.* 226.8 cases per week or 9.1 *vs.* 76.8 *vs.* 98.5 cases per 100 000 population, respectively (Table 3). Overall trend in weekly data on COVID-19 incidence rate generated by interrupted time-series analysis. It revealed that the series has a unit root with more than one trend tested by Dickey−Fuller test (MacKinnon approximate *P*-value = 0.36) as shown in Figure 2b. This trajectory is concordant with the joinpoint regression analysis (Fig. 2a) and is corresponding to the time points at which state policies/ordinances introduced at week 3 (social distancing), week 8 (EROP 1), week 11 (EROP 2) and week 17 (a peak of COVID-19), and the facemask or covering requirement in business and social settings that was implemented on 12 July 2020 (Fig. 2b). Regarding influence of the EROP 1 and EROP 2 on COVID-19 incidence perspective, Figure 3 illustrated trends in changes of COVID-19 incidence and points of interest were estimated (Table 4).

**Table 3.** Impact of EROP policies on COVID-19 cases and incidence rate

Intervention	Effect	COVID-19 cases		COVID-19 incidence rate	
		Mean (SD)	Range	Mean (SD)	Range
EROP 1	Pre-	20.9 (10.3)	5–36	9.1 (4.5)	2.2–15.6
	Post-	176.9 (170.4)	6–600	76.8 (74.0)	2.6–260.6
EROP 2	Pre-	18.6 (9.8)	5–36	8.1 (4.2)	2.2–15.6
	Post	226.8 (165.0)	19–600	98.5 (71.6)	8.3–260.6

EROP, economic reopening; SD, standard deviation.

**Table 4.** Estimates from the interrupted times-series analysis of economic reopen business and COVID-19 incidence rate

Measure of interest	Model 1: 3/15/2020–8/29/2020		Model 2: 3/15/2020–7/11/2020	
	Estimate (95% CI)	*P*-value	Estimate (95% CI)	*P*-value
Intercept (*β*0)	8.66 (0.31–17.01)	0.04	8.66 (−0.75, 18.07)	0.07
Pre-intervention 1 (pre-EROP 1, *β*1)	0.13 (−1.86, 2.12)	0.89	0.13 (−2.11, 2.37)	0.90
Level change immediately after EROP 1 (*β*2)	1.20 (−6.47, 8.87)	0.75	1.20 (−7.45, 9.85)	0.77
Difference trend between pre-EROP 1 and EROP 1 (*β*3)	−3.38 (−5.60, −1.20)	0.004	−3.38 (−5.84, −0.93)	0.011
Difference trend between the EROP 1 and EROP 2 (*β*5)	10.93 (2.82–19.04)	0.01	38.91 (11.76–66.07)	0.009
EROP 1 trend (*β*_1_ _+_*β*_3_)	−3.25 (−3.91, −2.59)	<0.0001	−3.25 (−3.99, −2.50)	<0.0001
EROP 2 trend (*β*_1 +_ *β*_3 +_ *β*_5_)	7.68 (−0.32, 15.68)	0.059	35.66 (8.40–62.92)	0.015
Predicted value for counterfactual model (EROP 1 + EROP 2)	11.83 (−28.84, 52.50)	0.55	10.91 (−19.39, 41.20)	0.45
Predicted value for intervention of EORP 1 and EROP 2	141.75 (69.12–214.38)	0.001	175.45 (61.02–289.88)	0.006
Difference trend between pre-EROP 1 and EROP 2 (*β*_3 +_ *β*_5_)	7.55 (−0.69, 15.79)	0.07	35.53 (8.18–62.88)	0.016
Difference between predicted and counterfactual	129.91 (62.24–197.59)	0.001	164.55 (39.83–289.26)	0.014

COVID-19, coronavirus disease 2019; CI, confidence intervals; EROP, economic reopen; pre-EROP, pre-intervention of EROP.

The pre-EROP trend in weekly COVID-19 incidence rate per 100 000 persons was unchanged with a slope of 0.13 (*P* = 0.89) with a starting value (intercept) of 8.66 (95% CI 0.32–17.01). The COVID-19 rate was not changed immediately by influence of EROP 1 after its introduction (*β*^2^ = 1.20, *P* = 0.75; model 1) and (*β*^2^ = 1.20, *P* = 0.77; model 2) but COVID-19 incidence rate changed significantly after introducing the EROP 2 in both slope and post-EROP 2 trend. Predicted incidence of COVID-19 trend increased by 141.75 (95% CI 69.12–214.34) and by 175.45 (95% CI 61.02–289.88) cases per 100 000 persons per week. Compared to the estimated counterfactual rate, EROP 1 and 2 accounted for increases of 129.91 (95% CI 62.24–197.59) and 164.55 (95% CI 39.83–289.26) cases per 100 000 persons per week for the entire study period of 24 weeks (model 1) and the study of 17 weeks (model 2), respectively (Table 4).

### COVID-19 trajectories in Smith County and other counties in Texas

**Fig. 4.**
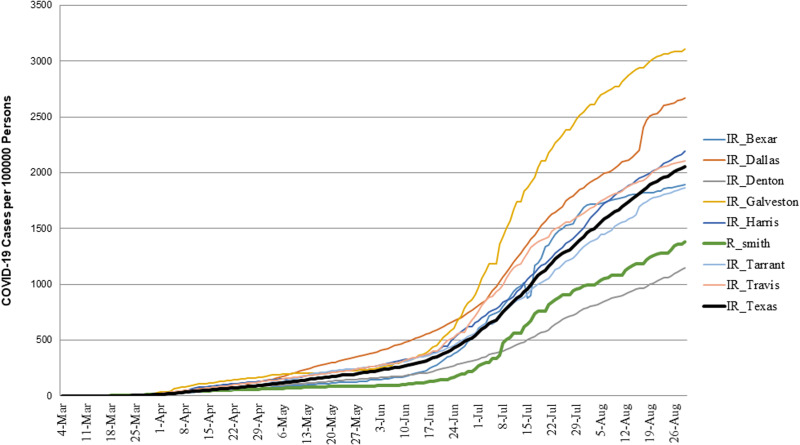
Comparison of daily cumulative incidence rate in selected counties in Texas. The figure shows a comparison of COVID-19 incidence rate in Smith County (green curve) and compared to other counties in Texas, including rural and urban counties, and overall incidence COVID-19 trajectory in State of Texas (black curve).

Inspection of the trajectory of COVID-19 incidence in Smith County and seven other counties, including rural and urban countries, as well as the state of Texas (Fig. 4). Visual comparison revealed that COVID-19 incidence rate in Smith County (1377.9 per 100 000) is slightly higher than Denton County (1149.5 per 100 000) but lower compared with other counties, Tarrant (1863.2 per 100 000), Bexar (1895.3 per 100 000), Travis (2106.7 per 100 000), Harris (2194.0 per 100 000), Dallas (2668 per 100 000), Galveston (3110.2 per 100 000) and overall Texas (2058.0 per 100 000).

## Discussion

The decisions to phase in business and societal reopening amid the pandemic has an infection cost that can be reduced or mitigated by high levels of community concordance with social distancing, facial covering in social settings, hand hygiene and good sanitation practices. The significant evidence in this study is the analysed difference between predicted infection rates without reopening *vs.* the predicted infection rate after reopening phases. Our study of COVID-19 incidence compared to economic reopening phases revealed 4 time periods that suggest varying levels of community concordance, associated with infection rates, as based on guidelines set forth in the reopening phased plans. There is not a value judgement on the actions and events that impact the COVID-19 infection rate as stated before, the whole social fabric or the plurality of the situation had to be considered. However, each action, those that enhanced community concordance and those that relaxed community concordance, had an impact on infection rate. Figure 3 shows COVID-19 incidence rate trend after the first phase EROP to increase as predicted. During phase 1, depending on the rurality of the county and having >5 active cases, services not otherwise considered essential (except bars, beauty/tattoo salons, gyms, public swimming pools and interactive amusement venues) re-opened at 25% capacity in the State of Texas [26]. Per the Governor's executive order, Texas citizens who accessed essential or non-essential services were advised to follow the health protocol set forth by DSHS [20, 22, 26]. In brief, the health protocol for individuals consists of wearing a facemask, maintaining 6 feet of social distancing, wash/sanitising hands frequently, self-screening for COVID-19 symptoms and avoiding gatherings of >10 people [17]. The extent that the individuals followed the recommended health protocol could explain the difference in predicted *vs.* actual COVID-19 cases. According to Seale *et al*. (2020), there is a multitude of psychological, demographic and social factors that impact engagement and compliance with non-pharmacologic interventions to slow the spread of a pandemic [27].

After phase 2 EROP opening as depicted in Figure 3, an even wider gap is seen between predicted cases and actual cases (higher). Per the executive order for phase 2 reopening, expansions of services and capacity were allowed and again recommendations to follow the DSHS health protocols were advised (EO-GA 23) [28]. Phase 2 reopening occurred at the beginning of June 2020 and within the subsequent 4–6 weeks other factors may have also contributed to community discordance rates to cause a higher than predicted number of COVID-19 cases such as the 4 July holiday weekend and ‘Black Lives Matter’ protests [29]. Incidentally, the governor issued a face mask mandate on 2 July; however, that may have been too close to the holiday weekend to effect behaviour change for planned 4 July celebrations and gatherings as the incidence rate peaked during the week of 5 July through 11 July [EO-GA-29] [30]. The mask mandate may have accounted for the downward trend after the peak as seen in Figures 2a and 2b. As evidenced by Al-Hasan *et al*. (2020), government plays a key role in positive adherence to non-pharmacologic interventions [31].

Non-pharmaceutical interventions such as movement management, social distancing, restriction on public gatherings and facemask or covering requirement in business and social settings were examined in this study to effectively mitigate the risk of COVID-19 transmission and for ‘flatten the curve’ of the pandemic in the Smith County. These non-pharmaceutical interventions have affirmed to be helpful in mitigating the risk of COVID-19 globally.

### Advantages and limitations

The main strength of this study was to utilise the joinpoint regression, an efficient and flexible statistical method, to determine the change points in time at which trends in COVID-19 incidence changed significantly in different periods. Additionally, interrupted time-series analysis is considered a quasi-experimental design that is a powerful tool used for evaluating the impact of interventions or policies implemented on outcomes of interest while randomised control trial data are not available.

This study has limitations. First, effects of interventions implemented may occur with unpredictable time delays, so we used a lag of 7 days. Poorly specified intervention points are overcome by using a Joinpoint regression analysis to identify joinpoints at which significant changes in trends in COVID-19 incidence occurred during the pandemic period studied along with the dates when the EROP interventions implemented. Second, the number of COVID-19 cases could be higher than confirmed cases depending on the number of tests conducted. Reported COVID-19 cases were limited according to testing capacity, speed and accuracy; however, we reported a mean with 95% confidence intervals. Third, population of Smith County and other counties used in this analysis were based on U.S. Census from 1 July 2019, which does not reflect actual population in March–August 2020; however, estimated COVID-19 cases and incidence are not much different because of small numerators of COVID-19 cases compared to big denominators of study populations. Fourth, the weakness of the ITSA is possibly impacted by something else that may have occurred at the same time such as the 4 July holiday weekend and ‘Black Lives Matter’ protests; however, trends in COVID-19 incidence rate predicted were not different under influencing by the EROP 1, EROP 2, or their synergistic effect as shown in Figure 3. Furthermore, two models were tested (Table 4) for two different scenarios in which exogenous factors such as the 4 July holiday weekend and ‘Black Lives Matter’ protests were taken into account. Many families desired to get together for special occasions and take possible precautionary measures to evade COVID-19 during such family events or gatherings. However, an unknown in such events or gatherings is whether the participants are homogeneously immune to the infectivity or heterogeneously risky to contract the virus. Amid less clarity, of interest might be how best one can predict the number of COVID-19 cases after the union. In other words, a research goal for the analysts of infectious diseases is to address the similarities *vs.* differences between the primary and secondary groups in the union. The research goal appears simple and easy on the surface but is actually very complicated and challenging as pointed out in a recent article by Ioannidis *et al*. [32].

## Conclusion

Economic reopening policies influenced the COVID-19 incidence rate along with natural trends. However, the facial covering or mask mandate may have accounted for the downward trend after the peak as seen in the holiday weekend of 4 July. Community concordance with mitigation efforts are salient to the decision to reopen sectors of a county's economic and social businesses and organisations; the analyses illustrated the significant findings in a semi-rural county. The findings of our study regarding non-pharmaceutical interventions have potential implications to mitigate the risk of COVID-19 globally.

## Data Availability

The data that support the findings of this study are openly available in the websites of the Texas Health and Human Services (DSHS) COVID-19 Data at https://dshs.texas.gov/coronavirus/additionaldata/, reference number [17].
